# Safety assessment of cenobamate: real-world adverse event analysis from the FAERS database

**DOI:** 10.3389/fphar.2024.1369384

**Published:** 2024-03-15

**Authors:** Shihao Chen, Wenqiang Fang, Linqian Zhao, Huiqin Xu

**Affiliations:** Department of Neurology, The First Affiliated Hospital of Wenzhou Medical University, Wenzhou, China

**Keywords:** FAERS database, cenobamate, adverse drug events, epilepsy, real-world study

## Abstract

**Objective:**

This study aims to analyze adverse drug events (ADEs) associated with cenobamate from the FAERS database, covering the third quarter of 2020 to the second quarter of 2023.

**Methods:**

Data related to cenobamate-associated ADEs from the third quarter of 2020 to the second quarter of 2023 were collected. After standardizing the data, various signal quantification techniques, including ROR, MHRA, BCPNN, and MGPS, were employed for analysis.

**Results:**

Among 2535 ADE reports where cenobamate was the primary suspected drug, 94 adverse reactions involving 11 different System Organ Class (SOC) categories were identified through the application of four signal quantification techniques. More specifically, neurological disorders and injuries resultant from complications are frequent adverse reactions associated with cenobamate.

**Conclusion:**

Our research findings align with established results, affirming the favorable safety profile of cenobamate. Effective prevention of adverse reactions induced by cenobamate can be achieved through the establishment of efficient blood concentration monitoring and dose adjustments.

## 1 Introduction

Epilepsy, as a prevalent neurological disorder, is characterized by sudden abnormal discharges of brain neurons, leading to transient cerebral dysfunction and significantly impacting the physical and mental wellbeing as well as daily life of affected individuals. According to the 2019 Global Burden of Disease (GBD) study, epilepsy affects over 50 million people worldwide (Shu et al., 2023). Despite the fact that the majority of epilepsy patients can achieve seizure control through pharmacological intervention, a subset of patients exhibits poor responsiveness to existing antiepileptic drugs (Panebianco et al., 2023). Hence, the urgent need to identify more effective and less adverse-reactive antiepileptic drugs persists.

The third-generation antiepileptic drug, cenobamate, received approval from the U.S. FDA in November 2019. Its primary mechanisms involve blocking sodium ion channels and positively modulating GABA receptor activity, exhibiting antiepileptic effects. Currently, both the FDA and EMA have sanctioned its use for the treatment of focal epilepsy. Clinical studies demonstrate that, compared to other antiepileptic drugs, cenobamate significantly excels in reducing focal epilepsy seizures (Makridis and Kaindl, 2023). Furthermore, several real-world studies substantiate its significant benefits in treating many drug-resistant epilepsy patients (Beltran-Corbellini et al., 2023; Schmitz et al., 2023). Despite the broad therapeutic potential of cenobamate in managing epilepsy, attention should be directed towards its safety.

The FDA Adverse Event Reporting System (FAERS) serves as a platform for collecting and analyzing drug adverse events (ADEs) related to drug utilization (Iyer et al., 2014). These data represent a crucial resource for evaluating drug safety and effectiveness. The purpose of this article is to analyze adverse event signals related to cenobamate in the real-world using data mining techniques, providing insights for the clinical use of the drug.

## 2 Methods

Using the trade name “XCOPRI” as the search term in the U.S. FAERS database, we retrieved ADEs reports related to cenobamate from the third quarter of 2020 to the second quarter of 2023. Descriptions and classifications of ADE reports were based on the Preferred Term (PT) and System Organ Class (SOC) concentrated in the Medical Dictionary for Regulatory Activities (MedDRA) terminology set (version 24.0) released by the International Conference on Harmonisation of Technical Requirements for Registration of Pharmaceuticals for Human Use.

ADE reports primarily implicating cenobamate were selected, and duplicates were excluded to minimize bias in ADE risk signal identification. This study employed four methods for ADEs signal mining, including the Reporting Odds Ratio (ROR) method, the Medicines Healthcare Products Regulatory Agency (MHRA) method, Bayesian Confidence Propagation Neural Network (BCPNN) method, and Multi-Item Gamma Poisson Shrinker (MGPS) method (Sakaeda et al., 2013). The ROR method originated from the Lareb laboratory of the Dutch Pharmacovigilance Centre, characterized by less bias and higher sensitivity, hence it is widely applied (Moore et al., 2005). The MHRA method is an extension of the PRR method, combining the PRR value, absolute report numbers, and chi-square values on the premise of ensuring a minimum combination of cases. It is known for its high sensitivity and stability of results and is currently extensively used by the Medicines and MHRA of the United Kingdom (Rothman et al., 2004; Hou et al., 2014). However, studies have shown that the sensitivity of this method decreases as the number of reports increases (Zhang et al., 2017). At present, the BCPNN method is a mature signal detection technique applied both domestically and internationally. It is capable of early signal detection even with fewer data or in case of missing data, and its detection results become more stable as the number of reports increases (van Puijenbroek et al., 2000), but the method is computationally complex and lacks transparency. Additionally, the MGPS method has the advantage of detecting signals for rare events (Jiang et al., 2024). Although there is no gold standard for signal detection methods, each method has its characteristics, with respective advantages and disadvantages in terms of applicability and feasibility in the database. Consequently, this study employed a combination of four methods to obtain signals with strong associations. These four methods compare the ratio of target AEs for the target drug to the ratio of target AEs for all other drugs. If this ratio exceeds a set threshold, it is deemed imbalanced, indicating the generation of potential AEs signals. In this study, a positive signal for drug-related AEs is considered when at least one of the four algorithms meets the criteria; when all four algorithms meet the criteria, it suggests a strong association of AEs, thereby avoiding potential false-positive signals. The parameters required for the ROR and other formulas are calculated based on a 2 × 2 contingency table, which is specifically available in Table 1. Specific formulas and signal detection criteria for the four algorithms can be found in Table 2 (Bate et al., 1998; Evans et al., 2001; van Puijenbroek et al., 2002; Sakaeda et al., 2013).

**TABLE 1:** Fourfold table for calculation, used for comparing the association between a specific drug and the occurrence of a specific adverse event.

	Cenobamate-related ADEs	Non-cenobamate-related ADEs	Total
Cenobamate	a	b	a + b
Non-cenobamate	c	d	c + d
Total	a + c	b + d	N = a + b + c + d

ADE, adverse drug events. a is the number of cases where a specific adverse event occurred after using cenobamate, b is the number of cases where cenobamate was used but the specific adverse event did not occur, c is the number of cases where the specific adverse event occurred without the use of cenobamate, d is the number of cases where neither cenobamate was used nor the specific adverse event occurred.

**TABLE 2:** Four main algorithms are used to evaluate the correlation between cenobamate and AEDs. This includes ROR, MHRA, BCPNN, and EBGM methods, formulas, and thresholds.

Method	Formula	Threshold
ROR	$ROR=\frac{\left(a/c\right)}{\left(b/d\right)}=\frac{ad}{bc}$	a>3 and 95% CI (lower limit) > 1
	$SE\left(\ln ROR\right)=\sqrt{\left(\frac{1}{a}+\frac{1}{b}+\frac{1}{c}+\frac{1}{d}\right)}$	
	$95\%CI=e^{\ln\left(ROR\right)\pm 1.96\sqrt{\left(\frac{1}{a}+\frac{1}{b}+\frac{1}{c}+\frac{1}{d}\right)}\;}$	
MHRA	$PRR=\frac{a/\left(a+b\right)}{c/\left(c+d\right)}$	a>3, PRR>2 and χ^2^ > 4
	$x^{2}=\frac{{\left(\left|ab-cd\right|-\frac{N}{2}\right)}^{2}\times N}{\left(a+b\right)\left(c+d\right)\left(a+c\right)\left(b+d\right)}$	
BCPNN	$IC=\log_{2}\frac{a\left(a+b+c+d\right)}{\left(a+b\right)\left(a+c\right)}$	IC025 > 0
	$\gamma =\gamma ij\frac{\left(N+\alpha \right)\left(N+\beta \right)}{\left(a+b+\alpha i\right)\left(a+c+\beta j\right)}$	
	$E\left(IC\right)=\log_{2}\frac{\left(a+\gamma ij\right)\left(N+\alpha \right)\left(N+\beta \right)}{\left(N+\gamma \right)\left(a+b+\alpha i\right)\left(a+c+\beta j\right)}$	
	$SD=\sqrt{V\left(IC\right)}$	
	$IC025=E\left(IC\right)-2SD$	
MGPS	$EBGM=\frac{a/\left(a+b+c+d\right)}{\left(a+c\right)\left(a+b\right)}$	EBGM05 > 2
	$95\%CI=e^{\ln\left(EBGM\right)\pm 1.96\sqrt{\left(\frac{1}{a}+\frac{1}{b}+\frac{1}{c}+\frac{1}{d}\right)}\;}$	

N, the number of reports; a is the number of cases where a specific adverse event occurred after using cenobamate, b is the number of cases where cenobamate was used but the specific adverse event did not occur, c is the number of cases where the specific adverse event occurred without the use of cenobamate, d is the number of cases where neither cenobamate was used nor the specific adverse event occurred; ROR, reporting odds ratio; γ, γ_ij_ represent the parameters of the Dirichlet distribution; α, α_i_, β, β_j_ represent the parameters of the Beta distribution; SD, standard deviation; MHRA, healthcare products regulatory agency; BCPNN, bayesian confidence propagation neural network; MGPS, Multi-Item Gamma Poisson Shrinker; PRR, proportional reporting ratio; EBGM, empirical bayes geometric mean; χ2, chi-squared; IC, information component; IC025, the lower limit of 95% CI, for the IC; E(IC), the IC, expectations; V(IC), the variance of IC; EEBGM05, the lower limit of the 95% CI, for EBGM.

We used SPSS software version 26.0 (IBM, United States), Microsoft Excel 2019, and R software version 4.3.1 for statistical analysis. The creation of figures relied on the “ggplot2” package in the R language.

## 3 Results

### 3.1 Descriptive analysis

Following the exclusion of duplicates, data from reports logged between the third quarter of 2020 and the second quarter of 2023 were extracted from the FAERS database. Among 2,535 reports, cenobamate was identified as the primary drug used. The specific relevant information and calculated figures are provided in Supplementary Material S1. The majority of these reports originated from the United States (*n* = 2,378), with the United Kingdom contributing the second-highest number (*n* = 29). Within the pool of reports, a cumulative total of 770 serious ADEs were recorded, encompassing instances of fatalities, life-threatening outcomes, disability, and permanent damage. Of these, 315 reports indicated ADEs necessitating hospital admission, 375 reports noted other significant medical events of severity, and there were 36 reports marked with fatalities.

### 3.2 Signal detection

**FIGURE 1:**
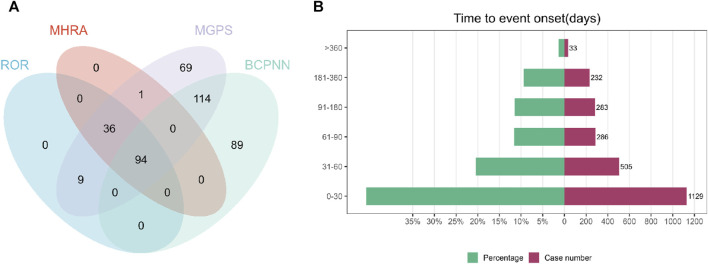
**(A)** The meticulous application of four distinct methodologies culminated in the identification of 94 efficacious PTs. Out of an assemblage of 882 signals, the ROR method surfaced 139 relevant signals, the MHRA method segregated 131, the EBGM method segregated 323, and the BCPNN method segregated 295 effective signals. **(B)** Onset Time of Adverse Reactions Related to Cenobamate.

Using four distinct algorithms, including the ROR method and BCPNN method, 139 PTs were found using the ROR method, 131 PTs were separated using the MHRA method, 323 PTs were separated using the EBGM method, and 295 PTs were separated using the BCPNN method. Ultimately, a total of 94 effective PTs were identified, as detailed in Figure 1A. The most prevalent PTs included Seizure (*n* = 648), Product Dose Omission Issue (*n* = 446), and Fatigue (*n* = 340). The top 30 PTs with the strongest associations is displayed in Table 3, according to the frequency of occurrence, while the detailed information for all positive signals is available in Supplementary Table S2. Furthermore, we probed the onset times of each PTs, as depicted in Figure 1B. It was observed that the PTs predominantly clustered within the first month post-medication (*n* = 1,129), thereafter exhibiting a decremental pattern over time. This insight could hasten the recognition and governance of safety issues related to cenobamate, thereby enabling prompt modifications in therapy to mitigate adverse reactions and augment the effectiveness of the treatment.

**TABLE 3:** The top 30 signal strength of adverse events of cenobamate ranked by number of incidence cases at the PTs level in FAERS database.

PTs	SOC	Case reports	ROR (95% CI)	PRR	EBGM	EBGM05	IC025
Seizure	Nervous system disorders	648	52.52 (48.45–56.93)	48.27	29,817.45	27,871.31	3.92
Product dose omission issue	Injury, poisoning and procedural complications	446	20.89 (18.99–22.99)	19.76	7,943.02	7,331.75	2.63
Fatigue	General disorders and administration site conditions	340	3.58 (3.22–4.00)	3.47	605.79	553.13	0.13
Somnolence	Nervous system disorders	334	13.39 (12.00–14.94)	12.86	3,659.10	3,338.16	2.02
Dizziness	Nervous system disorders	268	4.27 (3.78–4.82)	4.16	647.31	584.56	0.39
Fall	Injury, poisoning and procedural complications	155	3.66 (18.99–22.99)	3.61	293.89	257.27	0.19
Feeling abnormal	General disorders and administration site conditions	130	4.03 (3.39–4.79)	3.98	291.21	251.88	0.33
Gait disturbance	General disorders and administration site conditions	121	4.86 (4.06–5.82)	4.80	364.89	313.97	0.60
Wrong technique in product usage process	Injury, poisoning and procedural complications	110	4.70 (3.89–5.67)	4.65	315.39	269.42	0.55
Balance disorder	Nervous system disorders	109	9.65 (7.98–11.66)	9.53	831.92	710.12	1.58
Memory impairment	Nervous system disorders	85	4.78 (3.86–5.92)	4.74	251.26	210.10	0.58
Hypersomnia	Nervous system disorders	76	21.08 (16.81–26.43)	20.88	1,434.68	1,187.19	2.71
Product use issue	Injury, poisoning and procedural complications	74	3.28 (2.61–4.12)	3.26	115.96	95.74	0.04
Vision blurred	Eye disorders	70	4.07 (3.22–5.15)	4.04	160.44	131.76	0.35
Diplopia	Eye disorders	69	21.24 (16.75–26.93)	21.06	1,314.43	1,077.64	2.73
Lethargy	Nervous system disorders	57	7.54 (5.81–9.78)	7.49	320.51	257.69	1.24
Generalised tonic-clonic seizure	Nervous system disorders	55	28.86 (22.13–37.65)	28.67	1,462.33	1,170.72	3.17
Product availability issue	Product issues	51	28.41 (21.56–37.44)	28.23	1,334.12	1,059.04	3.15
Dysarthria	Nervous system disorders	46	9.32 (6.97–12.46)	9.27	339.16	266.07	1.54
Adverse event	General disorders and administration site conditions	46	3.89 (2.91–5.20)	3.88	98.26	77.09	0.29
Therapy interrupted	Surgical and medical procedures	43	8.47 (6.27–11.43)	8.43	281.20	218.79	1.41
Aura	Nervous system disorders	33	141.87 (100.41–200.46)	141.28	4,496.20	3,366.85	5.44
Disturbance in attention	Nervous system disorders	33	4.57 (3.25–6.44)	4.56	91.73	68.90	0.52
Feeling drunk	General disorders and administration site conditions	32	32.01 (22.60–45.33)	31.88	952.52	711.86	3.32
Irritability	Psychiatric disorders	31	3.82 (2.68–5.43)	3.81	64.18	47.77	0.26
Amnesia	Nervous system disorders	30	3.42 (2.39–4.89)	3.41	51.11	37.86	0.10
Speech disorder	Nervous system disorders	29	4.20 (2.92–6.05)	4.19	70.37	51.86	0.40
Anger	Psychiatric disorders	28	6.06 (4.18–8.79)	6.04	117.79	86.34	0.93
Abnormal behaviour	Psychiatric disorders	27	4.81 (3.29–7.01)	4.79	81.04	59.07	0.59
Partial seizures	Nervous system disorders	26	40.56 (27.56–59.68)	40.43	993.43	45.59	3.66

PT, preferred term; SOC, system organ class; ROR, reporting odds ratio; PRR, proportional reporting ratio; CI, confidence interval; IC, information component; IC025, the lower limit of the 95% CI, for IC; EBGM, empirical bayes geometric mean; EBGM05, the lower limit of the 95% CI, for EBGM.

### 3.3 Signals of system organ class

The 94 positive signals of PTs were classified according to the MedDRA 24.0 version SOC, revealing that 11 organ systems are impacted by AEs associated with cenobamate. Table 4 elucidates the signal intensities of the cenobamate-linked AEs stratified by SOCs. The positive signals predominantly clustered within three SOCs, namely: Nervous System Disorders (*n* = 2069), Injury, Poisoning and Procedural Complications (*n* = 865), and General Disorders and Administration Site Conditions (*n* = 705), with the comprehensive details of the remaining SOCs available in Table 4. Specifically, neurological disorders along with injuries due to complications such as falls or cranial impacts are noted as common adverse reactions to cenobamate.

**TABLE 4:** The signal strength of ADEs of cenobamate at the SOC level in FAERS database.

System organ class	SOC code	Case reports
Nervous system disorders	10,029,205	2069
Injury, poisoning and procedural complications	10,022,117	865
General disorders and administration site conditions	10,018,065	705
Psychiatric disorders	10,037,175	199
Eye disorders	10,015,919	155
Surgical and medical procedures	10,042,613	78
Product issues	10,077,536	62
Musculoskeletal and connective tissue disorders	10,028,395	16
Investigations	10,022,891	15
Respiratory, thoracic and mediastinal disorders	10,038,738	11
Social circumstances	10,041,244	6

SOC, system organ class; ADE, adverse drug events.

## 4 Discussion

Cenobamate, as one of the latest antiepileptic drugs, is commonly employed for the treatment of focal seizures in adult patients, offering advantages such as lower cost and improved tolerability (Specchio et al., 2021; Laskier et al., 2023). Functioning not only as a blocker of voltage-gated sodium channels and a positive modulator of GABA receptors, cenobamate also activates the PI3K/Akt-CREB-BDNF pathway, leading to elevated anti-apoptotic factor levels and reduced pro-apoptotic factor levels. This induction inhibits apoptosis, thereby enhancing neuronal survival (Wicinski et al., 2021).

In terms of pharmacokinetic studies on cenobamate, research by Roberti et al. indicates its nonlinear pharmacokinetics. The recommended initial dose of cenobamate is 12.5 mg/day, titrated gradually to the target daily dose of 200 mg, with the possibility of increasing to a maximum of 400 mg/day based on clinical response (Roberti et al., 2021). Some central nervous system-related side effects are more prevalent, including drowsiness, dizziness, diplopia, and gait and coordination disturbances, particularly when the daily dose exceeds 300 mg (Roberti et al., 2021).

Concurrently, studies support the significant improvement in seizure control among adults with uncontrolled focal seizures when cenobamate is used as adjunctive therapy at a dose of 200 mg/day, with good tolerability (Chung et al., 2020; Smith et al., 2022).

Based on clinical trial experience, cenobamate exhibits minor side effects, primarily consisting of dizziness and drowsiness (Catalan-Aguilar et al., 2023; Villanueva et al., 2023). Considering that various neurological and psychiatric conditions are common ADEs) associated with antiepileptic drugs, our study results corroborate this conclusion. Additionally, in patients treated with cenobamate, our study identified high-frequency and strong-signal ADEs such as Seizure (*n* = 648, ROR = 52.52, IC025 = 3.92) and generalized tonic-clonic seizure (*n* = 55, ROR = 28.86, IC025 = 3.17), which may be linked to treatment failure with cenobamate. Past research has established a close correlation between antiepileptic drug efficacy and blood drug concentration: elevated concentrations increase toxicity and the likelihood of ADEs, while insufficient concentrations fail to control seizures (Alldredge, 1999).

Furthermore, we observed adverse signals such as Fall (*n* = 155, ROR = 3.66, IC025 = 0.19) and Head banging (*n* = 4, ROR = 53.16, IC025 = 4.05). Although some falls may be attributed to poorly controlled seizure symptoms (Jung et al., 2023), numerous studies indicate that less than half of falls and fractures are directly associated with seizures. Falls are also frequent among patients taking antiepileptic drugs (Leppik et al., 2017), posing greater risks and severe consequences, particularly in elderly individuals. However, in another literature on falls in the elderly from the FAERS database, we found that the ROR value for cenobamate is lower than that for common antiepileptic drugs (Zhou et al., 2022), suggesting a favorable effect of cenobamate. Additionally, antiepileptic medications may impinge upon the functionality of the nervous system, encompassing balance and coordination capabilities, thereby elevating the risk of cranial impacts. Although the incidence of head collisions under cenobamate therapy appears to be infrequent, we must nevertheless maintain vigilance regarding this adverse reaction. In summary, monitoring blood drug concentrations during clinical use of antiepileptic drugs is necessary and holds significance for dose adjustments in epilepsy patients. Additionally, our observations revealed that cenobamate may trigger certain skin conditions, such as pruritic rash, possibly due to drug-induced allergic reactions. While generally mild, these skin reactions may serve as precursors to severe allergic reactions (Zgolli et al., 2023). Thus, seeking timely help and advice from healthcare professionals for appropriate diagnosis and treatment is crucial.

The adverse effects of antiepileptic drugs can significantly encroach upon a patient’s quality of life, precipitating physical discomforts such as fatigue, dizziness, and visual disturbances; psychological health issues, including mood fluctuations and depression; as well as cognitive impairments characterized by diminished memory and attention. These detriments may lead to reduced medication adherence, a decline in quality of life, increased economic strain, limited vocational choices, and an intensified sensation of social isolation, as reported in the literature (Kowski et al., 2016; Lin et al., 2016). Furthermore, patients who reduce or discontinue medication due to adverse reactions may experience escalated risks of epilepsy symptom recurrence (Shinnar and Berg, 1995; Ramos-Lizana et al., 2010). This scenario can result in a pernicious cycle that severely compromises the quality of life for many individuals living with epilepsy. To break this cycle, it is imperative to identify antiepileptic medications with fewer adverse reactions and minimal impact on quality of life. Our research observed that severe outcomes comprised 30.4% of the total reports, which signifies that cenobamate has achieved commendable results in clinical therapy, suggesting it might be a preferable treatment option.

Overall, this study, based on the FAERS database and utilizing the ROR method and PRR, among other algorithms, comprehensively presents the safety signal spectrum of cenobamate. It further substantiates cenobamate as a well-tolerated antiepileptic drug.

There are still some limitations in this study. Firstly, while the FAERS database boasts substantial volume and broad coverage, it is marred by incomplete data, with some reports lacking critical information such as age and gender. Additionally, as reporting is voluntary, there is an inherent risk of underreporting, delayed reporting, and misreporting of incomplete information, which introduces potential bias. Secondly, the utilization of analytical methods such as the ROR and PRR can only elucidate the association strength between the medication and ADEs, and cannot directly confirm causality. The actual relationship requires corroboration with existing literature and clinical application. Furthermore, our current study investigated only one limited safety dataset, with all reports predominantly originating from European and American countries. Given regional and ethnic variabilities, these findings may not be extrapolated to other populations, such as those in Asia. Lastly, given cenobamate’s relatively recent introduction to the market, larger-scale clinical trials in the future may unearth additional potential adverse signals. Hence, clinicians should remain vigilant regarding drug safety and promote the judicious use of cenobamate.

## 5 Conclusion

Our study, predicated upon the data derived from the FAERS database, indicates that cenobamate exhibits a commendable safety profile. We have deliberated on the preventive potential of adverse reactions associated with cenobamate, which can be effectively actualized through the establishment of vigilant therapeutic drug monitoring and meticulous dosage titration. These insights proffer substantive guidance for the clinical utilization of cenobamate in the treatment of epilepsy, further buttressing the assurance of patient safety and therapeutic efficacy during the administration of cenobamate.

## Data Availability

The original contributions presented in the study are included in the article/Supplementary Material, further inquiries can be directed to the corresponding author.
